# Probiotic Supplementation in a *Clostridium difficile*-Infected Gastrointestinal Model Is Associated with Restoring Metabolic Function of Microbiota

**DOI:** 10.3390/microorganisms8010060

**Published:** 2019-12-29

**Authors:** Mohd Baasir Gaisawat, Chad W. MacPherson, Julien Tremblay, Amanda Piano, Michèle M. Iskandar, Thomas A. Tompkins, Stan Kubow

**Affiliations:** 1 School of Human Nutrition, McGill University, 21111 Lakeshore, Ste. Anne de Bellevue, Montréal, QC H9X3V9, Canada; mohd.gaisawat@mail.mcgill.ca (M.B.G.); michele.iskandar@mail.mcgill.ca (M.M.I.); 2 Rosell Institute for Microbiome and Probiotics, 6100 Royalmount Avenue, Montréal, QC H4P 2R2, Canada; cmacpherson@lallemand.com (C.W.M.); apiano@lallemand.com (A.P.); ttompkins@lallemand.com (T.A.T.); 3 Energy, Mining and Environment, National Research Council Canada, 6100 Royalmount Avenue, Montreal, QC H4P 2R2, Canada; julien.tremblay@cnrc-nrc.gc.ca

**Keywords:** human gut microbiota, *Clostridium difficile*, probiotics, gastrointestinal model, short chain fatty acids, hydrogen sulfide, ammonium, 16S rRNA gene amplicon sequencing

## Abstract

*Clostridium* (*C.*) *difficile*-infection (CDI), a nosocomial gastrointestinal disorder, is of growing concern due to its rapid rise in recent years. Antibiotic therapy of CDI is associated with disrupted metabolic function and altered gut microbiota. The use of probiotics as an adjunct is being studied extensively due to their potential to modulate metabolic functions and the gut microbiota. In the present study, we assessed the ability of several single strain probiotics and a probiotic mixture to change the metabolic functions of normal and *C. difficile*-infected fecal samples. The production of short-chain fatty acids (SCFAs), hydrogen sulfide (H_2_S), and ammonia was measured, and changes in microbial composition were assessed by 16S rRNA gene amplicon sequencing. The *C. difficile*-infection in fecal samples resulted in a significant decrease (*p* < 0.05) in SCFA and H_2_S production, with a lower microbial alpha diversity. All probiotic treatments were associated with significantly increased (*p* < 0.05) levels of SCFAs and restored H_2_S levels. Probiotics showed no effect on microbial composition of either normal or *C. difficile*-infected fecal samples. These findings indicate that probiotics may be useful to improve the metabolic dysregulation associated with *C. difficile* infection.

## 1. Introduction

*Clostridium* (*C.*) *difficile* infection (CDI) is a toxin-mediated gastrointestinal (GI) disorder that is the leading cause of nosocomial infections [1]. CDI usually manifests as diarrhea, and in more severe cases, colonic inflammatory lesions and pseudomembrane formation [2]. An important factor in the pathogenesis of CDI is the presence of an altered gut microbial profile, which is strongly associated with antimicrobial therapy [3]. The GI concentrations of commensal microbes are decreased during CDI, which was shown to alter the colonic fermentative production of short chain fatty acids (SCFAs) [4,5]. Such metabolic disturbances could be important since SCFAs possess antimicrobial action, are critical in regulating immune function, and, in the case of butyrate, maintain intestinal cellular function and serve as a source of energy for colonic mucosal cells [4,5]. Furthermore, *C. difficile*-produced toxins A and B have demonstrated the potential to upregulate inflammatory pathways and induce cellular damage [6,7,8]. Probiotics have been considered as a therapeutic strategy to reduce the side effects of antibiotic therapy, counteract *C. difficile* growth, and reduce CDI-associated diarrhea [9,10].

Probiotics, defined as live microorganisms that impart beneficial effects on the host when given in adequate quantities [11], have shown beneficial effects in the GI tract such as improving metabolic function [12,13,14,15], counteracting infections [16,17,18], regulating immune function, decreasing GI disorder symptoms [16,19,20,21], and potentially lowering the risk of developing colon cancer [22]. Most of the probiotics utilized to date are usually from the *Lactobacilli*, *Bifidobacteria*, and yeast (*Saccharomyces*) groups. The efficacy and proposed mechanisms of action of these microbes in regulating intestinal microbiota functions are generally strain-specific. Some probiotic strains are thought to produce antimicrobial metabolites such as bacteriocins, to lower the pH by generating hydrogen peroxide and SCFAs, or to restrict pathogenic growth by competing for essential nutrients and adherence onto the gut mucosal barrier [18,23,24,25,26,27]. Several probiotics may reduce CDI-associated diarrhea and prevent primary CDI formation using some of the abovementioned mechanisms, but perhaps predominantly by inhibiting the adhesion of *C. difficile* in the intestine [28,29]. In the case of *Saccharomyces (S.) boulardii*, the mechanism was shown to involve the proteolytic hydrolysis of the CD enterotoxins A and B [30]. Although the *Lactobacillus (L.) rhamnosus* GG strain and *S. boulardii* have been studied the most in the context of CDI-associated diarrhea [9,31], several other strains such as *Bifidobacterium (B.) longum* and *L. acidophilus* CL1285 have also shown efficacy against antibiotic-associated diarrhea [32,33,34,35]. Furthermore, strains such as *L. plantarum 299v* have been shown to enhance microbial function in CDI patients receiving antibiotic treatment by increasing butyrate and total SCFA production [13].

Despite the potential benefits of some probiotics in the management of CDI, much remains to be elucidated concerning their ability to combat *C. difficile* infection and its associated changes to the gut microbiota. In this study, we assessed the effects of several probiotic strains, individually or in combination, on CDI microbiota in an in vitro gut model system. In vitro GI models have been validated for the simulation of gut microbiota and its associated metabolic functions such as production of SCFAs and gaseous by-products such as ammonium (NH_4_) and hydrogen sulfide (H_2_S) [36,37]. Several studies have shown disruption in the metabolic capacity of gut microbiota within several gastrointestinal disorders and following exposure to certain medications such as antibiotics [38]. Moreover, microbial alterations can lead to proliferation of certain bacterial groups such as sulfate-reducing bacteria, leading to a dysregulation of the metabolic capacity and abnormal levels of NH_4_ and H_2_S. Altered production of these latter gases and SCFAs has been implicated in several gastrointestinal complications such as disrupted metabolism of intestinal cells [39], and disease states such as inflammatory bowel disorders and colorectal cancer [40]. In that regard, probiotic supplementation has shown the capacity to enhance production of SCFAs [12,41] and to help in restoring overall metabolic capacity through regulation of the microbiota [25,42]. Furthermore, such models have been previously utilized by our research group to study the effect of digestion on biotransformation of polyphenols and anthocyanins along with their effects on SCFA production and metabolite toxicity on intestinal cells [43,44]. In the context of CDI, GI models have been utilized to study the efficacy of various antibiotics and their effect on *C. difficile* toxicity and commensal microbial communities [45]. The objective of the present study was to assess the changes in the metabolic function and microbial composition following *C. difficile* infection and determine whether probiotics could alleviate or minimize these changes. Individual strains *L. rhamnosus* R0011, *L. helveticus* R0052, *S. boulardii* CNCM I-1079, and *B. longum* R0175, along with a combination product, ProtecFlor^TM^ were studied for their efficacy. The study was conducted using an in vitro simulated GI model with *C. difficile*-infected fecal matter positive for both enterotoxins A and B. Metabolic function was assessed by quantification of microbial metabolites such as SCFAs, H_2_S, and NH_4_ [46]. Gut microbiota community structure of the fecal material from the fermentation experiments was assessed using 16S rRNA gene amplicon sequencing. Furthermore, detection of probiotic treatments was done using real-time polymerase chain reaction (qPCR) across time points to detect strain survivability.

## 2. Materials and Methods

### 2.1. Simulation of Gastrointestinal Conditions

This study utilized batch culture fermentation to simulate GI conditions as described previously [47]. Briefly, a dynamic computer-controlled model that houses 250 mL fermentation vessels was used to control for physiological colonic conditions such as pH, temperature, and an anaerobic environment. Each vessel was maintained at 37 °C using heated double-jacketed beakers and purged with oxygen-free nitrogen gas to maintain anerobic conditions. The pH was regulated continuously using an embedded EZO™ pH circuit (Atlas Scientific, Long Island City, NY, USA) controlled through a Raspberry Pi microprocessor (ver. 1B, Raspberry Pi Foundation, Cambridge, UK).

#### 2.1.1. Fecal Slurry Preparation

Fecal slurry was prepared as previously described [47]. Briefly, normal samples were obtained from a healthy adult male donor with no previous history of GI disorders and no antibiotic use within the past 6 months or more. Samples were diluted in 0.9% saline (1:3 *w*/*v*) and filtered using Whirl-Pak™ sterile filter bags (B01348WA, Thermo Fisher Scientific, Pittsburgh, PA, USA) followed by storage at −80 °C in a cryoprotectant solution (12.5% glycerol in 0.9% saline (*v*/*v*)) at a ratio of 1:3 *v*/*v*. *C. difficile* fecal samples were commercially sourced from BioIVT, Westbury, NY, USA (adult male; stool positive for enterotoxins A and B) and were processed in a similar manner. Regular fecal slurry was prepared by overnight stabilization of sample at 37 °C under anaerobic conditions. *C. difficile*-infected fecal slurry was prepared by adding *C. difficile* fecal slurry into the regular fecal slurry at a ratio of 1:10 *v*/*v* (5 mL of slurry per vessel).

#### 2.1.2. Probiotic Treatment Preparation

Four commercial single strain probiotic treatments and one multi-strain probiotic treatment were tested in this study. The single strain probiotics used were *L. rhamnosus* R0011 (R0011), *L. helveticus* R0052 (R0052), *S. boulardii* CNCM I-1079 (SB), and *B. longum* R0175 (R0175). ProtecFlor^TM^ (PROTO), a commercially available combination of R0011, R0052, R0175, and SB was used as the multi-strain probiotic. Probiotics were acquired from Lallemand Health Solutions Inc. (Montreal, QC, Canada) and stored at −20 °C until use. For inoculation in the fermentation vessel, each treatment was prepared by mixing the probiotic in sterile 1 × PBS and added at a dose of 1 billion cfu/vessel. Two treatment controls were used in this study: maltodextrin, the carrier base of the probiotic, was dissolved in 1 × PBS and used as vehicle control (hereinafter referred to as Vehicle), and 1 × PBS was used as the negative control (Blank).

#### 2.1.3. Batch Culture Fermentation

For batch culture fermentation, a modified method of Tzounis et al. (2008) [48] was used. Briefly, 100 mL of GI food, previously optimized by Molly et al. (1994) [49] (composed of 1 g/L of arabinogalactan, 2 g/L of pectin, 1 g/L of xylan, 3 g/L of starch, 0.4 g/L of glucose, 3 g/L of yeast extract, 1 g/L of peptone, 4 g/L of mucin, 0.5 g/L of cysteine, and 40 µL/L of vitamin solution; Sigma Aldrich, St. Louis, MO, USA) was added to each vessel. This was followed by a sequential enzymatic digestion in each vessel. Oral digestion was simulated by the addition of α-amylase (A3176, Sigma Aldrich, St. Louis, MO, USA) at pH 7.0 for 15 min, followed by stomach digestion by the addition of pepsin (P7125, Sigma Aldrich, St. Louis, MO, USA) at pH 2.0 for a period of 1.5 h, and pancreatic digestion by the addition of pancreatic juice (12 g/L NaHCO_3_, 6 g/L bile extract, and 0.9 g/L pancreatin; Sigma Aldrich, St. Louis, MO, USA) at pH 8.0 for 2 h.

After completion of enzymatic digestion, each vessel was inoculated with 50 mL of prepared regular or *C. difficile* infected fecal slurry (T = 0 h). Premixed probiotic treatment or blank (1 × PBS) was added to each vessel and fermentation was carried out under anerobic conditions with pH regulated at 6.3 ± 0.3 for a 24 h period with sampling after every 6 h. Samples were centrifuged at 2000× *g* for 10 min. The supernatant was filtered using sterile 0.45 µm syringe filters into new sample vials for metabolite analysis (hereinafter referred to as fecal water, FW). The fecal pellet was used for 16S rRNA gene amplicon community profiling and was stored at −80 °C until extraction. Each treatment (*n* = 7) was run in triplicate for both regular fecal slurry and *C. difficile*-infected fecal slurry batch culture fermentations.

### 2.2. FW Metabolite Analysis

#### 2.2.1. Short Chain Fatty Acids (SCFA) Analysis

SCFA analysis was conducted by a gas chromatograph system equipped with a flame ionization detector (GC-FID) (6890A series, Agilent Technologies, Santa Clara, CA, USA) using an adapted method outlined by Ekbatan et al. (2016) [43]. Briefly, 1 µL of 0.45 µm syringe filtered FW samples were directly injected into the GC-FID equipped with a fused capillary column (30 m × 250 µm ID × 0.25 µm film thickness; HP-INNOWAS, Agilent Technologies, Santa Clara, CA, USA). Helium was used as the carrier gas (1 mL/min). Inlet and detector temperatures were set at 220 and 230°C, respectively. For SCFA separation, the oven temperature was set at 100°C, held for 2 min followed by an increase of 10 °C/min until 220 °C where it was held for 1 min. Identification and quantification of individual SCFAs (acetate, propionate, butyrate, iso-butyrate, valeric acid, iso-valeric acid, caproic acid, iso-caproic acid, and heptanoic acid) was done using a free-volatile fatty acid standard (46975-U, Sigma Aldrich, St. Louis, MO, USA) and values were reported in mM as total SCFA, and as a combination of acetate, propionate, butyrate, and the remainder SCFA. Samples were analyzed in duplicate from each fermentation experiment.

#### 2.2.2. Hydrogen Sulfide (H_2_S) Determination Assay

Colorimetric determination of dissolved H_2_S in FW samples was conducted according to the zinc acetate precipitation method proposed by Gilboa-Garber (1971) [50]. Briefly, 0.5 mL of alkaline zinc acetate (2.6% *w*/*v* of zinc acetate and 6% *v*/*v* of sodium hydroxide mixed in a ratio of 5:1; Sigma-Aldrich, St. Louis, MO, USA) was added to 0.7 mL of FW sample. The mixture was centrifuged at 3000× *g* for 10 min to allow for precipitation of the zinc sulfide complex. After decanting the supernatant, the pellet was washed with 1.5 M sodium chloride (pH 8.0) and distilled water (pH 8.0). The pellet was then resuspended in 0.7 mL distilled water and vortexed, followed by the addition of 0.25 mL of *N, N*-dimethyl-*p*-phenylenediamine monohydrochloride (0.1% *w*/*v* in 5.5 N HCl; D5004, Sigma-Aldrich, St. Louis, MO, USA), and 0.1 mL of ferric chloride reagent (11.5 mM ferric chloride prepared with 0.6 N HCl; 157740, Sigma-Aldrich, St. Louis, MO, USA). The tubes were incubated at room temperature for 30 min for color formation to occur. An aliquot of 200 µL of solution was transferred into a 96-well microplate and absorbance was read at λ = 670 nm using a uQuant microplate reader (BioTek Instruments, Winooski, VT, USA). Seven equally distributed serial dilutions of 100 µM sodium sulfide were used to generate the analytical standard curve (R^2^ = 0.99). All samples were analyzed in triplicate from each independent fermentation experiment.

#### 2.2.3. Ammonium (NH_4_) Determination Assay

Colorimetric determination of NH_4_ was done using a microplate adapted method of the procedure outlined by Koroleff (1976) [51]. The procedure is based on the indophenol blue color formation when ammonium reacts with phenate in an alkaline solution in the presence of a strong oxidizing agent such as hypochlorite, and a metal-containing catalyst such as sodium nitroferricyanide (nitroprusside). Briefly, in a 96-well plate, 50 µL of FW sample or standard was added. This was followed by 25 µL of citrate reagent (0.2 M trisodium citrate in 0.5 M sodium hydroxide; 1110371000, Sigma Aldrich, St. Louis, MO, USA), 30 µL of salicylate-nitroprusside reagent (0.05 M sodium salicylate in 0.05 mM sodium nitroprusside; S3007 and 1614501, Sigma Aldrich, St. Louis, MO, USA), and 25 µL of hypochlorite reagent (10:2:1 *v*/*v*/*w* of household bleach, sodium hydroxide, and trisodium phosphate at pH 13.0). An aliquot of 145 µL distilled water was finally added to a total volume of 275 µL per well. The microplate was incubated at room temperature on a plate shaker for 30 min for complete color development. Absorbance was read at λ = 650 nm. An analytical standard curve (R^2^ = 0.99) was prepared using seven equally distributed serial dilutions of 36 mM ammonium sulphate (oven dried at 105 °C; A4418, Sigma Aldrich, St. Louis, MO, USA). Samples from each independent fermentation experiments were performed in triplicate.

### 2.3. DNA Extraction and Analysis

#### 2.3.1. DNA Extraction

Extraction of fecal DNA was done using the QIAamp® Fast DNA Stool Mini Kit (51604, Qiagen, Hilden, Germany) as per the manufacturer’s instructions. Prior to extraction, 300 to 500 mg of fecal pellet from each run were washed with 1 mL of 0.05 M phosphate buffer upon which InhibitEX (from the kit) and 0.1 mm zirconia beads (~300 mg/tube; 360991112, Thermo Fisher Scientific, Pittsburgh, PA, USA) were added. The sample tubes were then homogenized using a bead-beater (3 cycles of 4 m/s for 1 min; MP FastPrep®-24, MP Biomedicals, Irvine, CA, USA) followed by centrifugation at 13,000 rpm in a microcentrifuge for 3 min. DNA extraction was then carried out as per procedure outlined in the kit. Purity of extracted DNA was assessed by 260/280 ratios (absorbance at λ = 260 nm/280 nm) using NanoDrop™ One (Thermo Fisher Scientific, Pittsburgh, PA, USA). All DNA samples had ratios between 1.6–2.0. Samples were diluted in molecular-grade water to attain final concentrations of 20 ng/µL and stored at −20 °C prior to 16S rRNA gene amplicon sequencing.

#### 2.3.2. Detection of Probiotic Strains by Real-Time Polymerase Chain Reaction (qPCR)

Detection of individual probiotic strains, R0011, R0052, and R0175 was conducted in all extracted fecal DNA samples by real-time PCR (qPCR) once diluted five-fold in PCR-grade water. Strain-specific forward and reverse primers for R0011, R0052, and R0175 were obtained from Lallemand Health Solutions Inc. (Montréal, QC, Canada) and stored at −20 °C until use (Table 1). The qPCR assay specifications followed MIQE guidelines [52]. Each reaction consisted of 1X SYBR Select Master Mix (4472908, Applied Biosystems, Waltham, Massachusetts, USA), 300 nM of the respective forward and reverse primer, and 1 µL of template DNA. The 384-well qPCR assay plates were prepared by the epMotion 5075tc liquid handling robot (Eppendorf, Hamburg, Germany) by adding 9 µL of Mastermix and 1 µL of DNA to each well. Positive control DNA was extracted from pure overnight cultures of R0011, R0052, and R0175.

The following cycling conditions for each primer set (Table 1) were completed using the CFX384 Touch Real-Time PCR detection system (Bio-Rad Laboratories, Hercules, CA, United States): 50 °C for 2 min, followed by 95 °C for 2 min, followed by 40 cycles of denaturation at 95 °C for 15 s, annealing at 60 °C for 30 s, and extension at 72 °C for 30 s. A dissociation curve to ensure amplicon specificity was performed from 65 to 95 °C following the 40 cycles. The CFX Maestro^TM^ software (version 1.1, Bio-Rad Laboratories, Hercules, CA, USA) was used to perform the data analysis.

#### 2.3.3. 16S rRNA Gene Amplicon Sequencing and Bioinformatics

Characterization of microbial communities was performed by 16S rRNA gene amplicon sequencing as previously described [53]. Briefly, extracted DNA was used to construct sequencing libraries according to Illumina’s “16 S Metagenomic Sequencing Library Preparation” guide (Part # 15044223 Rev. B), with the exception of using Qiagen HotStar MasterMix for the first PCR (“amplicon PCR”) and halving reagent volumes for the second PCR (“index PCR”). The template specific primers were (without the overhang adapter sequence) the following: forward (5′-CCTACGGGNGGCWGCAG-3′) and reverse (5′- GACTACHVGGGTATCTAATCC-3′), targeting the V3-V4 hypervariable region [54] specific to bacterial organisms and generating a fragment of around 460 bp. The first PCR (“amplicon PCR”) was carried out for 25 cycles with annealing temperatures of 55 °C. Diluted pooled samples were loaded on an Illumina MiSeq system and sequenced using a 500-cycle (paired-end sequencing configuration of 2x250 bp) MiSeq Reagent Kit v3.

Sequencing data was analyzed using AmpliconTagger, the National Research Council of Canada’s amplicon pipeline [55]. Briefly, raw reads were scanned for sequencing adapters and PhiX spike-in sequences and remaining reads were merged using their common overlapping part with FLASH [56]. Primer sequences were removed from merged sequences and remaining sequences were filtered based on quality (Phred) score. Remaining sequences were clustered at 100% identity and then clustered/denoised at 99% identity (Vsearch v2.7.1, [57]). Clusters having abundances lower than three were discarded. Remaining clusters were scanned for chimeras with VSEARCH’s version of UCHIME denovo and UCHIME reference [57,58] and clustered at 97% (VSEARCH) to form the final clusters/operational taxonomic units (OTUs). A global read count summary is provided in Supplementary Table S1. OTUs were assigned a taxonomic lineage with the RDP classifier [59] using an in-house training set containing the complete Silva release 128 database [60] supplemented with eukaryotic sequences from the Silva database and a customized set of mitochondria, plasmid, and bacterial 16S sequences. The RDP classifier gave a score (0 to 1) to each taxonomic depth of each OTU. Each taxonomic depth having a score ≥0.5were kept to reconstruct the final lineage. Taxonomic lineages were combined with the cluster abundance matrix obtained above to generate a raw OTU table, from which a bacterial organisms OTU table was generated. Five hundred 1000 reads rarefactions were then performed on this latter OTU table and the average number of reads of each OTU of each sample was computed to obtain a consensus rarefied OTU table (available in Supplementary Table S2). A multiple sequence alignment was obtained by aligning OTU sequences on a Greengenes core reference alignment [61] using the PyNAST v1.2.2 aligner [62]. Alignments were filtered to keep only the hypervariable region of the alignment. A phylogenetic tree was built from that alignment with FastTree v2.1.10 [63]. Alpha (Shannon index) and beta (weighted UniFrac distances) diversity metrics and taxonomic summaries were then computed using the QIIME v1.9.1 software suite [62,64] using the consensus rarefied OTU table and phylogenetic tree (i.e., for UniFrac distance matrix generation).

### 2.4. Statistical Analyses

All data are reported as means ± standard error of mean (SEM). Data for SCFA, H_2_S, and NH_4_ were analyzed using two-way ANOVA using probiotic treatment (7 levels) and time (5 levels) as factors. For multiple comparisons, Dunnett’s post hoc test was carried out to compare treatments to control (blank). The means of all time points were jointly considered when no significant interactions in the two-way ANOVA were observed. When significant interactions between time and treatment were observed, the mean of each time point within a treatment was individually compared to its corresponding time point within the control along with Tukey’s HSD post analysis to assess for significant differences within treatment. Statistical significance was set at *p* < 0.05. All two-way ANOVA and post-hoc statistical analyses, and visualizations for metabolite data were performed using JMP v14.2 (SAS Institute, Cary, NC, USA). PERMANOVA analyses were done with R (v3.6.0) using the adonis2 function of the Vegan (v2.5-4) package. Taxonomic profiles, alpha- and beta-diversity plots were generated with R (ggplot2 v3.1.1).

### 2.5. Availability of Data

Raw sequence reads of the 16S rRNA gene amplicon data were submitted to the sequence read archive under Bio Project PRJNA565012.

## 3. Results

### 3.1. SCFA Determination in FW

**Figure 1:**
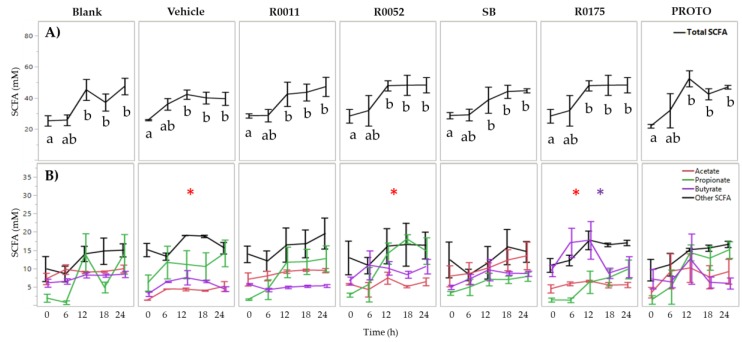
Short-chain fatty acid (SCFA) analysis of normal FW. (**A**) Total SCFA quantification and (**B**) individual SCFA quantification. Values are presented as the means ± SEM. Means at time points within treatments without a common letter are significantly different (*p* < 0.05). The symbol * in red represents significant differences in acetate production between treatment and blank (*p* < 0.05) when the means of all time points are jointly considered. The symbol * in purple represents significant differences in butyrate production between treatment and blank (*p* < 0.05) when the means of all time points are jointly considered. R0011 = *L. rhamnosus* R0011; R0052 = *L. helveticus* R0052; SB = *S. boulardii* CNCM I-1079; R0175 = *B. longum* R0175; PROTO = ProtecFlor^TM^.

Two-way ANOVA results for total SFCAs only showed a significant (*p* < 0.05) main effect of time for normal FW whereby time 0 h was significantly lower than time 12, 18, and 24 h. Supplementation with probiotics did not change the total SCFA levels in normal FW. The levels of acetate and butyrate, however, differed significantly (*p* < 0.05) amongst the probiotic treatments when compared to blank. The vehicle, R0052, and R0175 treatments showed significantly lower (*p* < 0.05) production of acetate whereas R0175 showed significantly higher (*p* < 0.05) production of butyrate (Figure 1).

**Figure 2:**
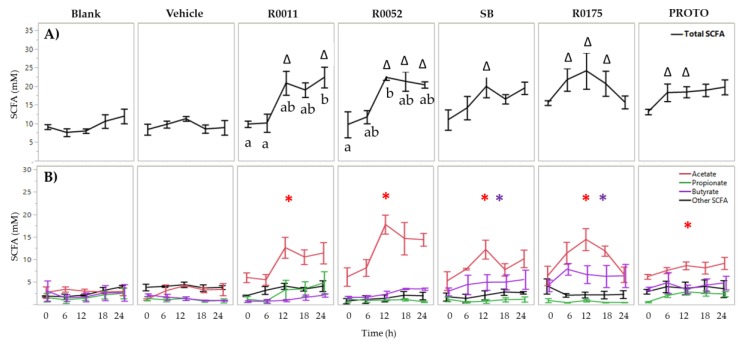
Short-chain fatty acid (SCFA) analysis of *Clostridium (C.) difficile*-infected normal fecal water. (**A**) Total SCFA quantification and (**B**) Individual SCFA quantification. Values are shown as mean ± SEM. The symbol ∆ represents significant differences (*p* < 0.05) between treatment at a particular time point and blank at the corresponding time point. Means at time points within treatments without a common letter are significantly different (*p* < 0.05). The symbol * in red represents significant differences in acetate production between treatment and blank (*p* < 0.05) when the means of all time points are jointly considered. The symbol * in purple represents significant differences in butyrate production between treatment and blank (*p* < 0.05) when the means of all time points are jointly considered. R0011 = *L. rhamnosus* R0011; R0052 = *L. helveticus* R0052; SB = *S. boulardii* CNCM I-1079; R0175 = *B. longum* R0175; PROTO = ProtecFlor^TM^.

In *C. difficile*-infected FW, two-way ANOVA results for total SCFA showed significant (*p* < 0.05) main effects of treatment, time, and an interaction effect of treatment and time. Therefore, the mean total SCFA for each time point within each treatment was compared to its corresponding time point of the blank. Each of the probiotic treatments showed a significant (*p* < 0.05) increase in total SCFAs. R0175 and PROTO showed a significant increase starting at time 6 h and at time 12 h. R0011 showed significantly (*p* < 0.05) higher total SCFAs at time 12 and 24 h whereas R0052 showed a significant (*p* < 0.05) increase at time 12, 18, and 24 h (Figure 2). The increase in total SCFA production for each of these probiotic treatments could be attributed to a significant (*p* < 0.05) increase in acetate production as compared to blank when the means of all time points were jointly considered. Furthermore, SB and R0175 showed a significantly (*p* < 0.05) higher butyrate production compared to the blank (Figure 2).

In order to determine the ability of probiotic supplements to produce SCFAs in GI food, a batch culture experiment for a 24 h period was performed without the presence of fecal slurry. R0011, R0052, SB, and PROTO showed significantly (*p* < 0.05) higher total SCFA production when compared to Vehicle (Figure S1), whereas R0175 showed no significant effect. This observed increase in SCFAs was principally due to significantly (*p* < 0.05) higher acetate levels in R0011, SB, and PROTO at 24 h. Moreover, significantly (*p* < 0.05) higher levels of butyrate were also observed in SB and PROTO cultures at 24 h (Table S1).

### 3.2. NH_4_ and H_2_S Determination in FW

**Figure 3:**
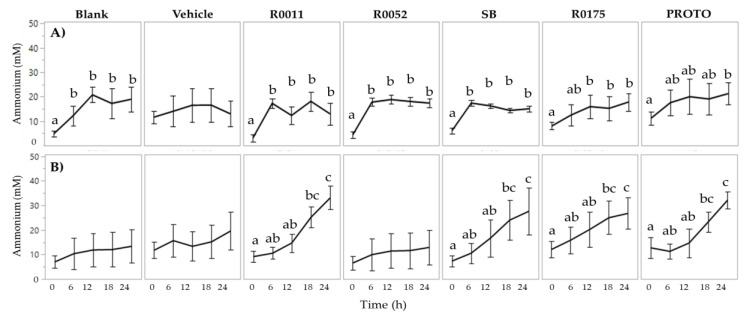
Determination of ammonium in fecal water (FW); (**A**) Normal FW and (**B**) *Clostridium* (*C.*) *difficile*-infected FW. Values are shown as mean ± SEM. Means at time points within treatments without a common letter are significantly different (*p* < 0.05). R0011 = *L. rhamnosus* R0011; R0052 = *L. helveticus* R0052; SB = *S. boulardii* CNCM I-1079; R0175 = *B. longum* R0175; PROTO = ProtecFlor^TM^.

Determination of NH_4_ in FW showed no effect of probiotic supplementation in both normal FW and *C. difficile*-infected FW. Two-way ANOVA results for ammonium showed a significant (*p* < 0.05) main effect of time for both normal FW and *C. difficile*-infected FW. In normal FW, time 0 h was seen to be significantly (*p* < 0.05) lower than all the other time points (6 to 24 h) for Blank, R0011, R0052, and SB. Similarly, time 0 h was significantly (*p* < 0.05) lower than time 24 h in PROTO, and, lower than time 12 to 24 h in R0175. In *C. difficile*-infected FW, time 0 h was significantly (*p* < 0.05) lower than time 18 h and time 24 h in R0011, SB, R0175, and PROTO (Figure 3). However, despite the observed differences in ammonia production over time, no significant effect of treatment was observed in both normal FW and *C. difficile*-infected FW.

**Figure 4:**
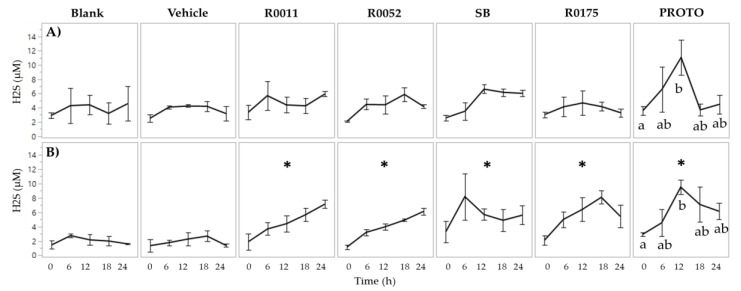
Determination of H_2_S in fecal water (FW); (**A**) Normal FW and (**B**) *Clostridium* (*C.*) *difficile*-infected FW. Values are shown as mean ± SEM. The symbol * represents significant differences between treatment and blank (*p* < 0.05) when the means of all time points are jointly considered. R0011 = *L. rhamnosus* R0011; R0052 = *L. helveticus* R0052; SB = *S. boulardii* CNCM I-1079; R0175 = *B. longum* R0175; PROTO = ProtecFlor^TM^.

Hydrogen sulfide production in normal FW showed no significant main effects of treatment. In *C. difficile*-infected FW, however, two-way ANOVA results showed significant (*p* < 0.05) main effects for both time and treatment. Furthermore, H_2_S levels were found to be lower in *C. difficile*-infected FW in comparison to normal FW, by 2.9-, 1.6-, 2.3-, 1.5-, and, 2.8-fold at times 0, 6, 12, 18, and 24 h, respectively. Supplementation with probiotics in *C. difficile*-infected FW resulted in a significant (*p* < 0.05) increase of H_2_S production (Figure 4). Moreover, PROTO showed a significantly (*p* < 0.05) higher H_2_S production at time 12 h compared to time 0 h in normal and *C. difficile*-infected FW.

### 3.3. Detection of Probiotic Strains by qPCR

**Figure 5:**
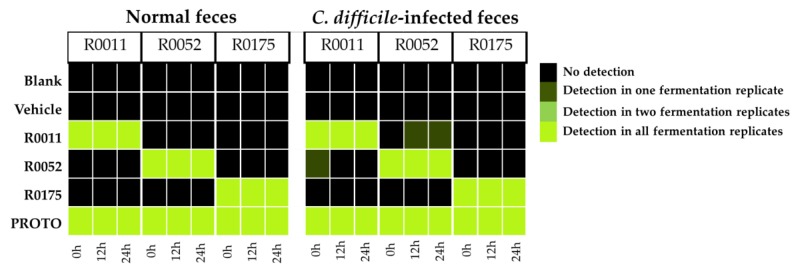
Detection of individual probiotic strains in extracted fecal DNA samples by real-time PCR (qPCR). Detection of strain *B. longum* R0175; Detection of strain *L. helveticus* R0052; Detection of strain *L. rhamnosus* R0011. Each column of the corresponding time point represents an individual experiment along with corresponding quantification cycle (Cq) value. R0011 = *L. rhamnosus* R0011; R0052 = *L. helveticus* R0052; R0175 = *B. longum* R0175; PROTO = ProtecFlor^TM^.

Detection of the bacterial strains R0011, R0052, and R0175 was conducted across all the fecal samples. Positive detection was confirmed by comparing the amplicon melt curve to the positive control in samples with a threshold quantification cycle (Cq) value less than 30. The results from the qPCR detection show that the strains were positively detected in their respective samples in normal feces and *C. difficile*-infected feces across all time points of batch fermentation. As each of the individual bacterial strains are present in the PROTO probiotic mix, all the strains showed positive detection in samples from that treatment. Some false positive qPCR detections for R0052 at the 18 and 24 h time points were observed in one of the R0011 replicates, as well as for R0011 at the 0 h time point for one of the R0052 replicates. These false positive detections could be due to non-specific binding of the primers to similar sequences from other *Lactobacilli* in the microbiota (Figure 5), as reported previously when detecting *Bifidobacterium* strains [65].

### 3.4. Microbial Community

16S rRNA gene amplicon sequencing was used to profile microbiota composition of fecal samples collected from batch culture fermentation at time 0, 12, and 24 h. Metrics such as alpha diversity (Shannon index), beta diversity (Weighted UniFrac) and relative abundance of observed species were used to characterize these microbial communities.

**Figure 6:**
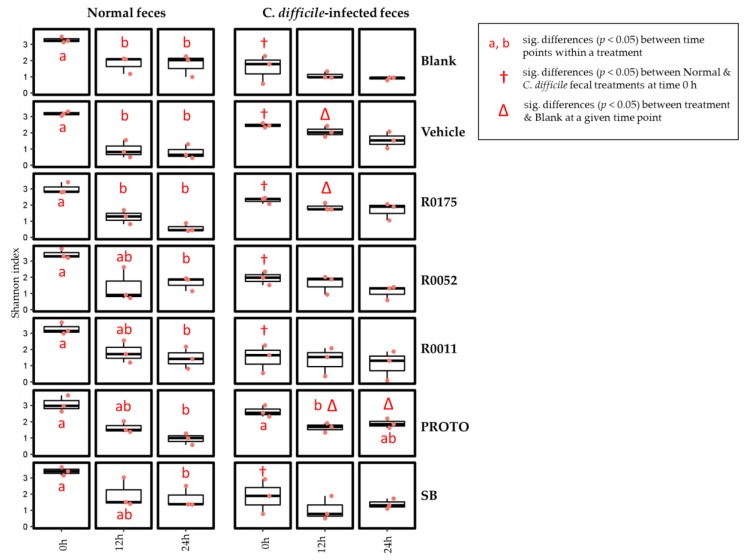
Microbial alpha diversity in normal and *Clostridium* (*C.*) *difficile*-infected feces assessed by the Shannon index. The symbol † represents significant (sig.) differences (*p* < 0.05) between feces for a particular treatment at time 0 h. The symbol ∆ represents significant differences (*p* < 0.05) between treatment at a particular time point and blank at the corresponding time point. Means at time points within treatments without a common letter are significantly different (*p* < 0.05). R0011 = *L. rhamnosus* R0011; R0052 = *L. helveticus* R0052; SB = *S. boulardii* CNCM I-1079; R0175 = *B. longum* R0175; PROTO = ProtecFlor^TM^.

Two-way ANOVA analysis followed by matched pairs Student’s t-test was conducted on Shannon index (alpha diversity) to assess for differences in microbial communities. To assess for differences between the fecal samples at time 0 h, pairwise comparisons showed that normal samples had an overall higher alpha diversity score as compared to *C. difficile*-infected samples, with all treatments except for PROTO showing a significant (*p* < 0.05) effect. The changes in microbial diversity within a given treatment was done by comparing the means of the time 12 and 24 h to the mean at time 0 h. The results for alpha diversity showed that in normal FW, there was a significant (*p* < 0.05) decrease in microbial species richness over time for all treatments. Blank, Vehicle, and R0175 showed a significant (*p* < 0.05) decrease starting at time 12 and 24 h, whereas R0011, R0052, PROTO, and SB showed a significant (*p* < 0.05) decrease only at time 24 h. In *C. difficile*-infected fecal samples, only PROTO showed a significant (*p* < 0.05) decrease at time 12 h (Figure 6). Differences in the microbial community richness (alpha diversity) between each treatment was done by comparing the values of a treatment at a particular time point to that of the blank at the corresponding time point. The results showed significant effects only in the *C. difficile*-infected fecal samples where Vehicle and R0011 showed significantly (*p* < 0.05) higher community richness at time 12 h, and PROTO showed a significantly (*p* < 0.05) higher diversity at time 12 and 24 h (Figure 6).

**Figure 7:**
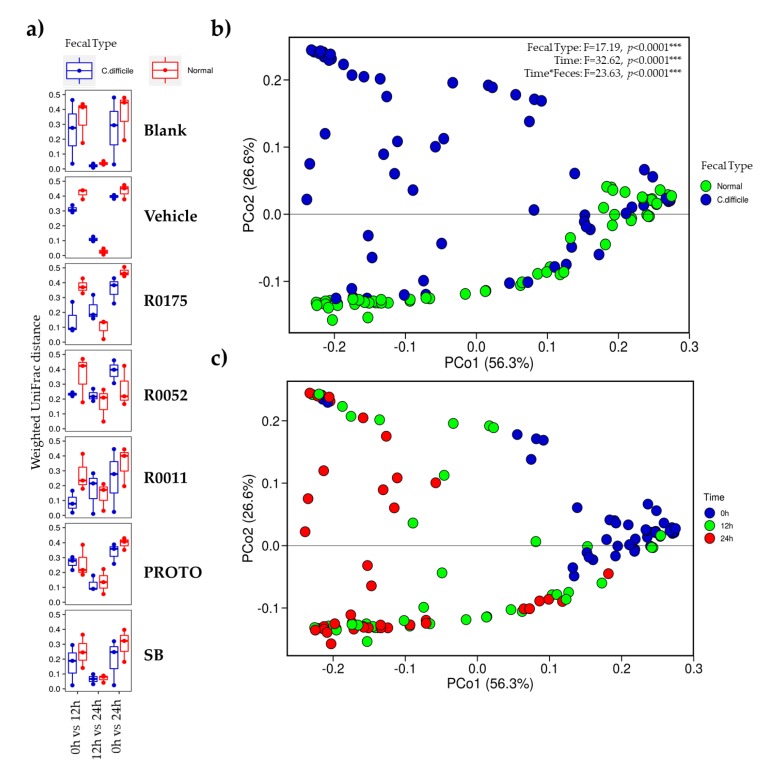
Beta diversity plots of normal fecal samples, and *Clostridium* (*C.*) *difficile*-infected fecal samples showing (**a**) Weighted UniFrac distance and PCA plots (**b**) clustered by type of feces, and (**c**) clustered by time. R0011 = *L. rhamnosus* R0011; R0052 = *L. helveticus* R0052; SB = *S. boulardii* CNCM I-1079; R0175 = *B. longum* R0175; PROTO = ProtecFlor^TM^.

The results of the beta diversity showed that there were differences in the similarity of the microbial community structures over time. Samples of all treatments in both normal and *C. difficile*-infected feces showed an overall higher similarity of the microbiota at time 12 h when compared to time 24 h, whereas the microbiota was relatively dissimilar at time 0 h when compared to time 24 h (Figure 7). This effect is less pronounced in samples of R0175, R0052, and R0011 when supplemented in *C. difficile*-infected feces, where there is less microbial community similarity when each of the time points were compared to each other. 

PERMANOVA analyses performed on weighted UniFrac distances (Figure 7) showed that samples cluster differed primarily by time and type of stool, indicating that these two variables are the main drivers in the formation of distinct communities. Interestingly, the normal fecal samples and *C. difficile*-infected fecal samples clustered together at time 0 h suggesting similar microbial community. The clustering of samples microbial communities was also observed in OTU heatmaps (Supplementary Figures S2 and S3), where the blanks of the respective fecal sample clustered at time 0 h.

**Figure 8:**
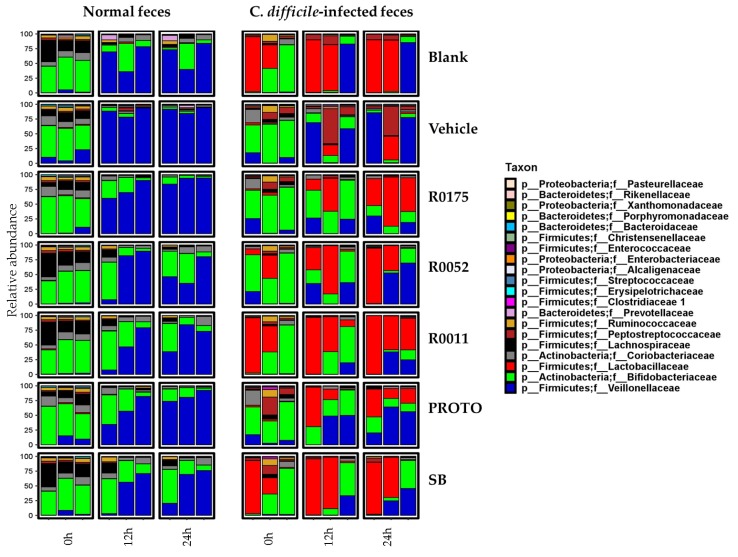
Taxonomic profiles (family level) of normal feces, and, *Clostridium* (*C.*) *difficile*-infected feces showing relative abundance over time. Values are shown in operational taxonomic units (OTUs). R0011 = *L. rhamnosus* R0011; R0052 = *L. helveticus* R0052; SB = *S. boulardii* CNCM I-1079; R0175 = *B. longum* R0175; PROTO = ProtecFlor^TM^.

Taxonomic profiles were generated to investigate microbial community structures across the experimental conditions. The relative abundance of the top 20 taxa down to the family level for both the fecal sample groups is shown in Figure 8. The results showed that the most prevalent taxa at the family level in normal fecal samples at time 0 h were of *Bifidobacteriaceae*, followed by *Lachnospiraceae* and *Coriobacteriaceae*. At time 12 and 24 h, however, the family *Veillonellaceae* becomes most abundant, followed by *Bifidobacteriaceae* for all treatments in normal fecal samples. In *C. difficile*-infected fecal samples at time 0 h, the taxa *Bifidobacteriaceae* and *Lactobacillaceae* were the most predominant, followed by *Peptostreptococcaceae* and *Coriobacteriaceae*. At time 12 and 24 h, *Lactobacillaceae* still show a high abundance, followed by an increase in abundance of *Veillonellaceae* and a decrease in abundance of *Bifidobacteriaceae*. Interestingly, the probiotic treatments were observed to have a higher proportion of *Bifidobacteriaceae* at time 12 and 24 h.

One of the primary objectives for this study was to assess the effects of probiotic supplementation on the microbial composition of the fecal samples. Overall, no effect of probiotic treatments was noted across time for either of the fecal slurry preparations (Figure 8). On the other hand, notable differences were seen in alpha and beta diversity in the *C. difficile*-infected fecal samples with probiotic supplementation (Figure 6; Figure 7). In that regard, PROTO showed increased alpha diversity at time 24 h compared with *C. difficile*-infected feces at the same time point, while the strains R0175, R0052, and R0011 appeared to show decreased changes in beta diversity in the *C. difficile*-infected feces. No major compositional changes were observed in the microbiota when comparing probiotic treatments to Blank or Vehicle (Figure 8). The above observations remained unchanged when the taxonomic profiles were assessed by amplicon sequence variants (ASVs) (Figure S5).

## 4. Discussion

The results from the present study showed that probiotic supplementation in *C. difficile*-infected fecal matter resulted in significant increases in the production of SCFAs and H_2_S. In terms of the microbial communities, however, no overall effect of probiotics was observed with respect to changes in microbial composition in *C. difficile*-infected fecal matter when compared to the controls. Metabolite analyses showed that the levels of total SCFAs in normal fecal samples were similar to previously reported literature values of 20–70 mM for the transverse and proximal colonic regions [66]. Probiotic supplementation in normal FW was associated with no overall alteration in total SCFA production, although some changes were observed in individual SCFAs. Vehicle, R0052, and R00175 showed lower production of acetate, and R0175 treatment resulted in higher production of butyrate when compared to Blank. These latter differences in SCFA profiles, but not in total SCFA production, can be speculated to be due to differences in microbial interactions between the treatment groups. The increase in total SCFAs over time for the normal fecal samples could be attributed to the increased presence of the family *Veillonellaceae* (Figure 8), particularly that of *Megaspheara* spp. (Supplementary Figure S1). *Megaspheara* spp. have been shown to produce a range of SCFAs in the human gut through fermentation of lactate and glucose substrates [67,68]. More specifically, glucose utilization by *Megaspheara* spp. has been associated with the production of acetate, caproate, butyrate, and isovalerate, amongst other SCFAs [67]. 

In contrast to normal fecal samples, the total production of SCFAs in *C. difficile*-infected fecal samples was significantly (*p* < 0.05) reduced in both the controls, ranging between 5–15 mM total SCFAs. This observation is in line with reported literature whereby patients with *C. difficile* infection show hampered production of SCFAs [4,69]. Supplementation with each of the probiotic treatments resulted in significantly (*p* < 0.05) higher total SCFA production when compared to controls, reaching nearly 30 mM total SCFA at time 24 h. As seen in Figure 2, this latter increase in total SCFAs can be attributed to the significant (*p* < 0.05) increase in overall acetate production. The probiotics SB and R0175 also showed a significant (*p* < 0.05) increase in butyrate production. The ability of *Lactobacilli* spp. and *Bifidobacteria* spp. to regulate and increase acetate production in the human gut has been well documented [70,71]. In a study by Sivieri et al. (2013), supplementation with *L. plantarum* in a GI model resulted in higher levels of all the major SCFAs, with the highest increase seen in acetate production [72]. Moreover, *S. boulardii* has been previously associated with an increase in total SCFAs and individual SCFAs such as acetate, propionate, and butyrate [41,73]. The above findings are further supported by the results of SCFA production by the probiotic supplements in GI food culture in the absence of fecal microbiota. All supplements, except R0175, showed a significant (*p* < 0.05) increase in total SCFAs compared to Vehicle (Figure S1). Moreover, R0011 showed a significant (*p* < 0.05) increase in acetate, and SB and PROTO showed significant (*p* < 0.05) increases in acetate and butyrate (Table S1). The latter results provide further support for the potential of these supplements to contribute to the overall production of SCFA in the gut microbiota, particularly with respect to the increased acetate and butyrate levels observed in *C. difficile*-infected fecal samples. It is interesting to note that *B. longum* R0175 supplementation in the *C. difficile*-infected fecal slurry resulted in significantly (*p* < 0.05) higher levels of butyrate. *Bifidobacteria* fall under the category of acetogens, i.e., they have been shown to produce mainly acetate through carbohydrate fermentation pathways [71]. Although they have generally not be been seen as capable of producing butyrate through fermentation, many studies have speculated that due to symbiotic cross-feeding interactions between *Bifidobacteria* and butyrate-producing colonic bacteria, such as *Faecalibacterium prausnitzii*, *Eubacterium*, and *Roseburia* spp., supplementation with *Bifidobacteria* could result in better survival of these bacteria and so lead to higher butyrate production [74,75].

Apart from products of carbohydrate fermentation, the protein fermentation products of NH_4_ and H_2_S were also measured to assess intestinal homeostasis. Each of these metabolites has been previously associated with changes in gut microbial composition and overall colonic health [39,76]. High levels of NH_4_ have been linked to cytotoxic effects on the gut lumen, contributing to the formation of colorectal cancer [77,78]. Similarly, H_2_S has been linked to a range of toxicity pathways [79,80]. The results from the present work show that NH_4_ production in the GI model was within the normal range of NH_4_ production in the human gut lumen [36,39]. Although the production of NH_4_ significantly (*p* < 0.05) increased in all samples compared to time 0 h, which is indicative of the fermentation process, the levels of NH_4_ thereafter remained stable with no statistical differences among timepoints. Furthermore, no effect of probiotics on NH_4_ production was observed in either normal or *C. difficile*-infected fecal samples, and no significant differences were found between the two fecal types. It is possible that these latter results could be due to the limitation in the sensitivity of the assay, or that the level of protein in the GI food was not sufficient enough to see changes in NH_4_ levels between the fecal types. With regards to H_2_S production, normal fecal samples showed no overall differences between the treatments and the levels of H_2_S were within the normal colonic range [40]. The *C. difficile*-infected fecal control samples, however, had lower levels of H_2_S when compared to normal fecal samples. These levels appeared to be restored to the level found in the normal fecal samples when supplemented with each of the probiotic treatments (Figure 4). The depletion of H_2_S observed in the *C. difficile*-infected fecal samples coincides with previous observations of inflammatory bowel conditions being associated with dysregulation of sulphate producing bacteria and disruption in some of the key functions of H_2_S such as colonic mucus production and maintenance of microbiota biofilm [79,80]. The ability of probiotic supplementation to increase and restore H_2_S levels in the *C. difficile*-infected fecal samples could be linked with the concurrent increased generation of acetate and butyrate. Production of acetate and butyrate by intestinal bacteria is thought to occur via the glycolytic pathway, which converts carbohydrates to pyruvate and acetyl-CoA. This latter process generates H_2_ as a byproduct, which thereafter undergoes sulfate reduction in the gut to form H_2_S [71].

16S rRNA gene amplicon sequencing was performed on all the samples to observe the changes occurring in the *C. difficile*-infected fecal samples and the possible shift in microbial communities during probiotic supplementation. These results showed that *C. difficile*-infected samples had a lower alpha diversity when compared to normal samples at time 0 h (Figure 6). Furthermore, relative abundance of the microbial communities showed that in both types of fecal samples, the richness in microbial diversity was not maintained across the time points, possibly arising from the batch culture conditions where poor microbiological control has been previously documented [37]. However, despite this limitation, normal samples had a more stable and richer microbiota when compared to *C. difficile*-infected samples at time 0 h (Figure 8 and Supplementary Figure S2), as observed in previous literature [3]. Additionally, normal samples showed little variation between fermentation replicates whereas *C. difficile*-infected replicates failed to reproduce similar microbial relative abundances at time 0 h. The variation in the *C. difficile*-infected fecal slurry at time 0 h could be attributed to its lower initial diversity when compared to normal samples. Such lesser diversity could have resulted in different microbial interactions and growth rates leading to poorer microbial control. The results of beta diversity plots, however, showed that at time 0 h, normal and *C. difficile*-infected fecal samples clustered together, showing similarities in their microbial community structure. The reason for no major differences in initial microbial community structure could be due to the resilience of the normal fecal microbiota to compositional changes in the absence of antibiotic treatment [81]. Despite beta diversity plots showing community similarity at time 0 h, the patterns across time 12 and 24 h differed with time and type of fecal sample. Normal fecal samples were closely clustered at each corresponding time point, whereas *C. difficile* samples showed scattering across those time points (Figure 6), indicating variations in microbial communities. This above result was confirmed with the relative abundance data (Figure 8), which showed variations in microbial groups such as *Lactobacillaceae*, *Veillonellaceae*, and *Bifidobacteriaceae* across treatments and time for the *C. difficile*-infected samples. Moreover, the strain R0175 and the probiotic mix PROTO seemed to show similar patterns of microbial communities (Figure 8) and were closely clustered in the heatmaps of each fecal type (Supplementary Figures S3 and S4), suggesting a possible dominant effect of R0175 in the mix. In the present study, however, no major shifts in microbial composition were observable when probiotics were supplemented in either fecal type. Similar observations were noted in previous studies; as shown by a study by Lahtinen et al. (2012) which demonstrated that *L. rhamnosus* HN001 and *L. acidophilus* NCFM were associated with changes to *Lactobacilli* and *C. difficile* but did not show any significant effects on major microbial groups [82]. Similarly, a study by Forssten et al. (2015) demonstrated that supplementation with *L. acidophilus* NCFM, and *L. paracasei* Lpc-37 did not show changes in the colonic microbiota in terms of reducing the *C. difficile* microbial population [83]. It has been suggested that this phenomenon could be due to slow growth rates of probiotics observed in the GI tract whist remaining metabolically active [25,84], thus explaining their inability to cause significant changes in the microbiota composition under batch culture conditions in the present study. Hence, inherent limitations of the batch culture design with respect to microbiological control could have masked the effects of the probiotics on the microbial communities in the fecal samples. 

## 5. Conclusions

To summarize, the results of the metabolite assays of the *C. difficile*-infected fecal samples collectively showed a range of changes, which indicated impaired key metabolic functions. Probiotic supplementation (R0011, R0052, SB, R0175, and, PROTO) increased SCFA levels and restored depleted H_2_S levels in *C. difficile*-infected fecal samples. In normal fecal samples, however, probiotics did not affect metabolic functions. Furthermore, 16S community profiling showed that normal fecal samples, across all treatments, had a closer similarity between its microbial communities at each time point, in contrast to *C. difficile*-infected fecal samples, which showed community similarity only at time 0 h signifying community disruption at time 12 and 24 h. Moreover, *C. difficile*-infected fecal samples displayed a lower diversity at time 0 h, in accordance with previous literature [3]. Despite the occurrence of strain-specific effects amongst the tested probiotics, such as the increase of microbial diversity by *B*. *longum* R0175 and ProtecFlor^TM^ at certain time points, no drastic shifts in the microbiota composition were observed in the *C. difficile*-infected samples. Similarly, probiotic supplementation did not affect microbiota composition in normal fecal samples.

In conclusion, the present work has revealed that using an in vitro gastrointestinal model, metabolic functions changes induced by *C. difficile* infection (CDI) in a fecal sample were measurable, as well as the effect of probiotics on overall microbiota diversity and their metabolic output. Supplementation with single strain probiotics (*L. rhamnosus* R0011, *L. helveticus* R0052, *S. boulardii* CNCM I-1079, *B*. *longum* R0175) and a probiotic mixture (ProtecFlor^TM^) restored microbial metabolic functions but was not associated with quantifiable changes in microbiota composition. Nevertheless, despite not having seen changes in *C. difficile*-infected microbiota in this model system, the metabolite analyses indicate the potential of probiotics to restore intestinal metabolic homeostasis, suggesting that they could be useful adjuncts to antibiotic therapy in CDI. Further research is warranted to establish the role of probiotics in restoring intestinal metabolic functionality in the context of CDI through the use of fecal samples from different population groups and from patients with different levels of CDI-pathophysiology.

## Figures and Tables

**Table 1:** Primer and target sequences for qPCR detection.

Bacterial Strain	Primer Name	Targeted Sequence	Gene Target	Amplicon Size (bp)
*B. longum* R0175	R175_AP_HP10_F	GTC GCC ACA TTT CAT CGC AA	Hypothetical protein	99
	R175_AP_HP10_R	GAG AGC TTC GAT TGG CGA AC		
*L. helveticus* R0052	pIR52-1-orf5 F1	AGA ATC AAG CAG AGA CTG GCT ACG	An ORF in a plasmid specific to R0052	150
	pIR52-1-orf5 R1	GGA CCG GAT TTG AGT AGA GGT A		
*L. rhamnosus* R0011	113A29_293FL	ACT CCA AAG AGC ATT ACC TCC G	113A29 phage head protein	71
	113A29_321RU	TGA ATA TGC CGG ATC TAA GTC CA		

## Supplementary Materials

The following are available online at https://www.mdpi.com/2076-2607/8/1/60/s1, Table S1. Reads count throughout key bioinformatics processing steps, Table S2. Consensus rarefied OTU table, Table S3. Short-chain fatty acid (SCFA) production by probiotics in gastrointestinal (GI) food media, Supplementary Figure S1. Total short-chain fatty acid (SCFA) production by probiotic supplementation in gastrointestinal (GI) food media, Figure S2. Microbial diversity (genus level) of normal feces, and *Clostridium* (*C.*) *difficile*-infected feces showing relative abundance over time, Figure S3. Heatmap of operational taxonomic units (OTUs) of normal fecal samples showing clustering over time, Figure S4. Heatmap of operational taxonomic units (OTUs) of *Clostridium* (*C.*) *difficile*-infected fecal samples showing clustering over time, Figure S5. Microbial diversity (family level) of normal feces, and *Clostridium* (*C.*) *difficile*-infected feces showing relative abundance using amplicon sequence variants (ASVs).
