# Supplementary material for: Probiotic Supplementation in a Clostridium difficile-Infected Gastrointestinal Model Is Associated with Restoring Metabolic Function of Microbiota
Source: Microorganisms. 2019 Dec 29;8(1):60. doi: 10.3390/microorganisms8010060 (PMC7023328; doi:10.3390/microorganisms8010060)
Supplement: Supplementary file 1 [file microorganisms-08-00060-s001.zip › Supplementary Final Version 19.10.2019.docx]

Article

Probiotic Supplementation in a *Clostridium difficile*-Infected Gastrointestinal Model is Associated With Restoring Metabolic Function of Microbiota

Mohd Baasir Gaisawat ^1^, Chad W. MacPherson ^2^, Julien Tremblay ^3^, Amanda Piano ^2^, Michèle M. Iskandar ^1^, Thomas A. Tompkins ^2^, Stan Kubow ^1^

Supplementary Data


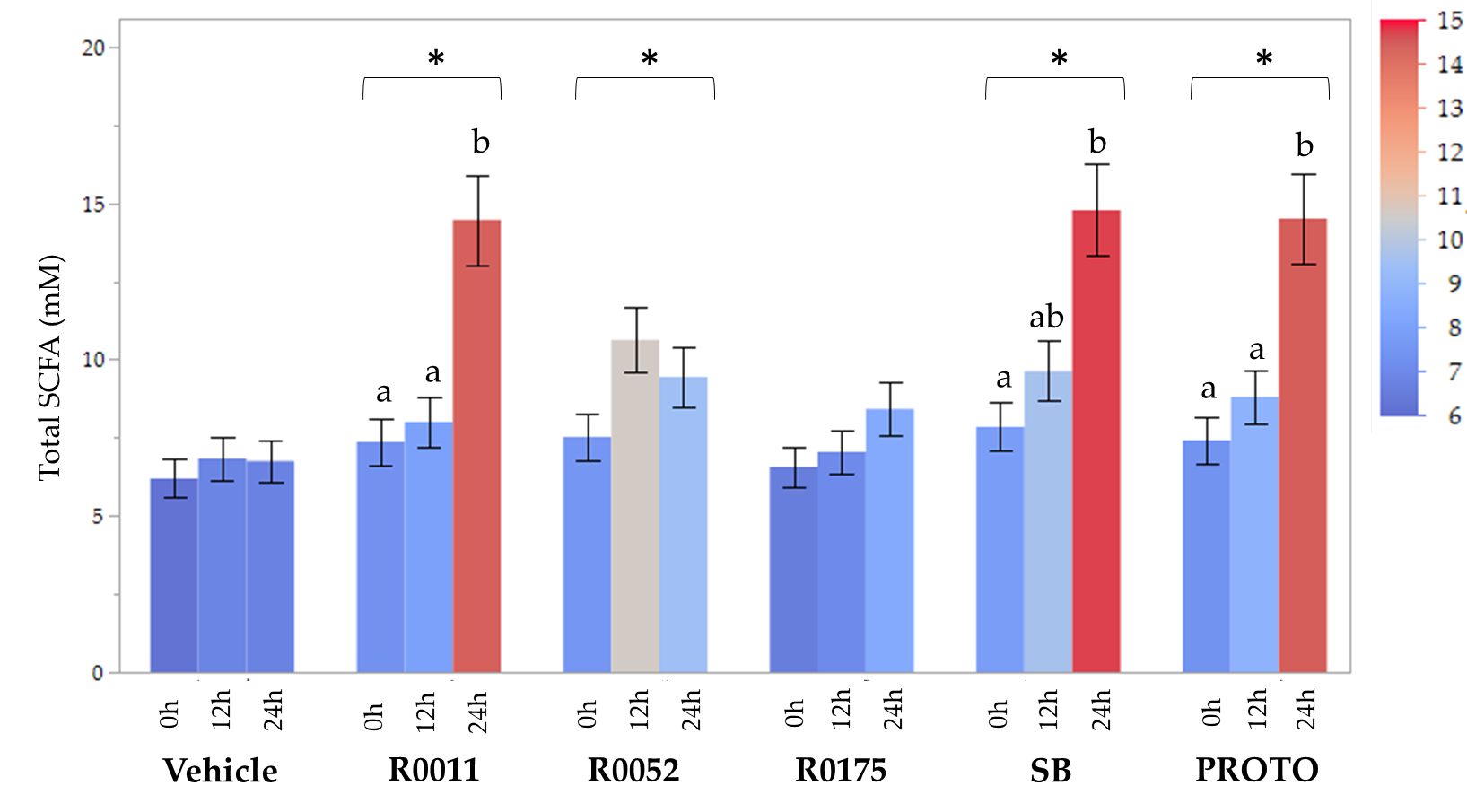


**Supplementary Figure S1.** Total short-chain fatty acid (SCFA) production by probiotic supplementation in gastrointestinal (GI) food media. Values are presented as means ± SEM. Means at time points within treatments without a common letter are significantly different (p < 0.05). The symbol * represents significant differences in SCFA production between treatment and control (p < 0.05) when the means of all time points are jointly considered. R0011 = *L. rhamnosus* R0011; R0052 = *L. helveticus* R0052; R0175 = *B. longum* R0175; SB = *S. boulardii* CNCM I-1079; PROTO = ProtecFlor^TM^.

**Table S3.** Short-chain fatty acid (SCFA) production by probiotics in gastrointestinal (GI) food media

| Treatment | Time | Acetate (mM) | Propionate (mM) | Butyrate  (mM) | Remainder SCFA (mM) |
| --- | --- | --- | --- | --- | --- |
| Control (Vehicle) | 0 h  12 h  24 h | 5.79  6.31  5.07 | 0.15  0.16  0.59 | 0.16  0.17  0.20 | 0.19  0.19  0.89 |
| *L. rhamnosus* R0011 | 0 h  12 h  24 h | 6.94 ^a^  7.02 ^a^  13.22 ^b^ | 0.18  0.19  0.76 | 0.10  0.16  0.10 | 0.15  0.63  0.38 |
| *L. helveticus* R0052 | 0 h  12 h  24 h | 6.96  9.31  8.62 | 0.22  0.19  0.20 | 0.17  0.21  0.17 | 0.17  0.92  0.45 |
| *B. longum* R0175 | 0 h  12 h  24 h | 6.25  6.66  7.98 | 0.12  0.14  0.18 | 0.09  0.08  0.14 | 0.10  0.17  0.12 |
| *S. boulardii* | 0 h  12 h  24 h | 7.45 ^a^  9.11 ^ab^  11.94 ^b^ | 0.15  0.18  0.18 | 0.07 ^a^  0.15 ^a^  2.43 ^b^ | 0.17  0.20  0.22 |
| Protecflor^TM^ | 0 h  12 h  24 h | 6.94 ^a^  8.25 ^ab^  12.46 ^b^ | 0.17  0.18  0.10 | 0.11 ^a^  0.12 ^a^  1.76 ^b^ | 0.19  0.24  0.18 |

Remainder SCFA include: iso-butyrate, valeric acid, iso-valeric acid, caproic acid, iso-caproic acid, and, heptanoic acid. Values are shown as mean.


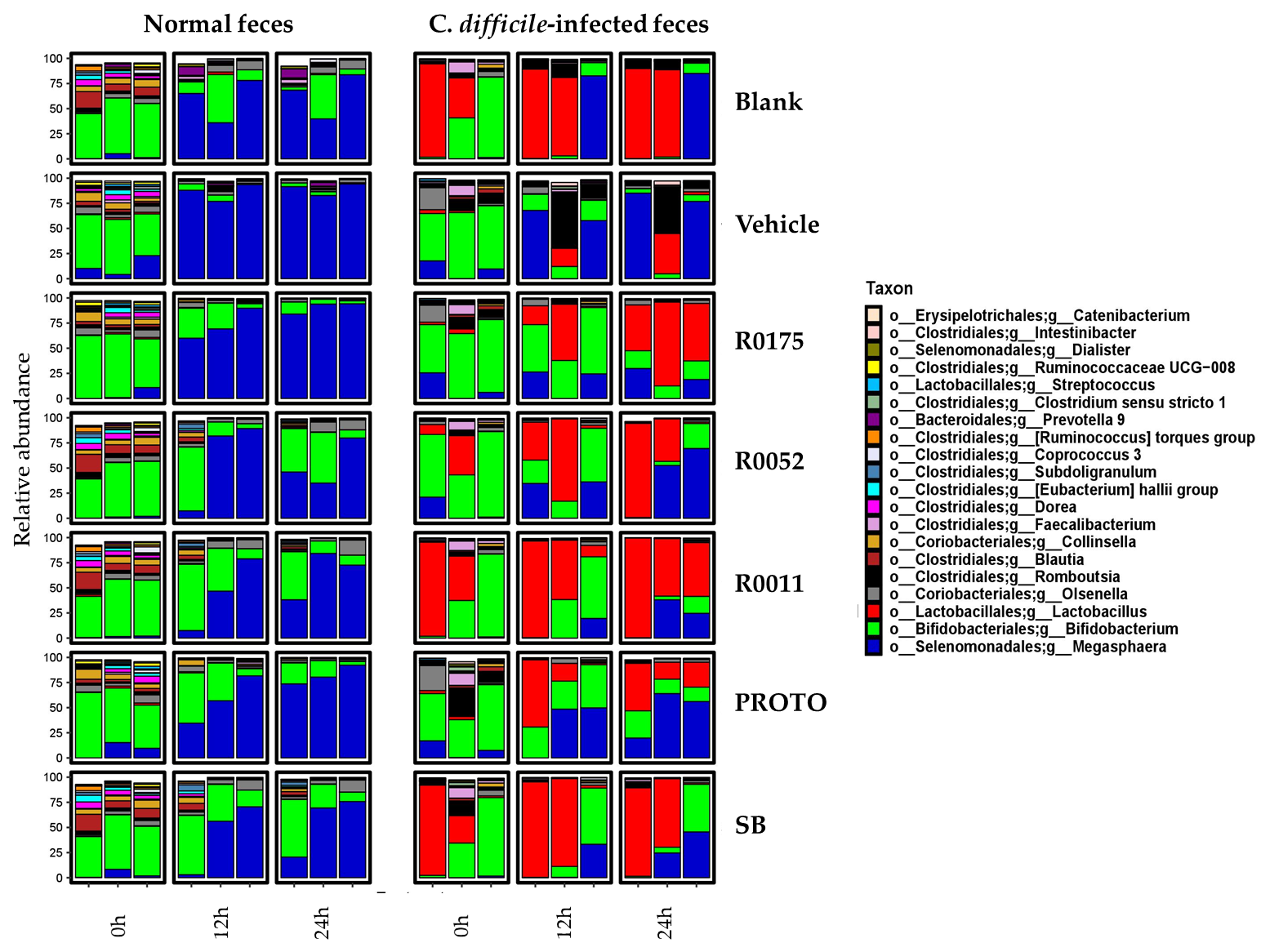


**Supplementary Figure S2.** Microbial diversity (genus level) of normal feces, and, *Clostridium* (C.) *difficile*-infected feces showing relative abundance over time. Values are shown in OTUs. R0011 = *L. rhamnosus* R0011; R0052 = *L. helveticus* R0052; SB = *S. boulardii* CNCM I-1079; R0175 = *B. longum* R0175; PROTO = ProtecFlor^TM^.


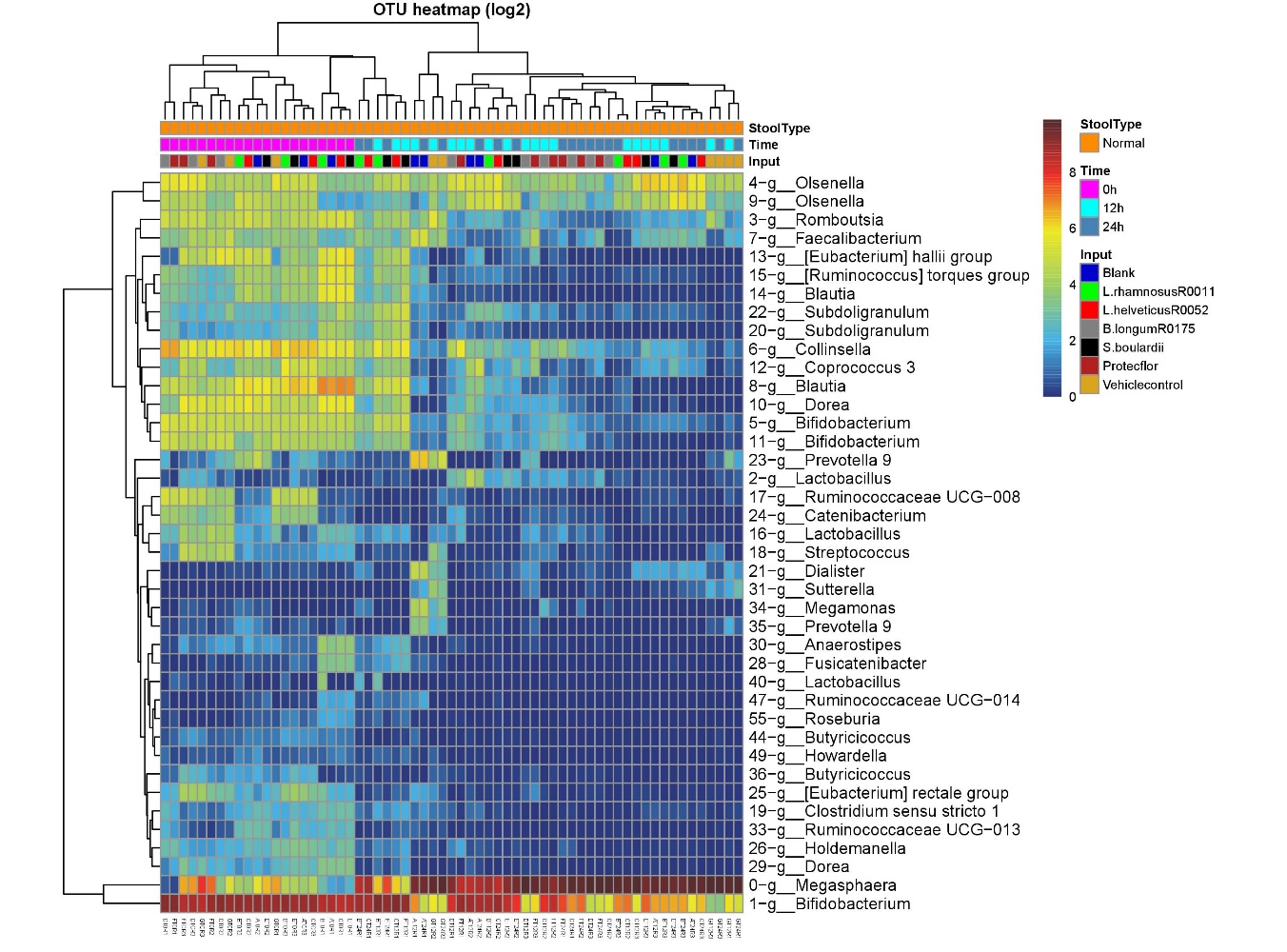


**Supplementary Figure S3.** OTU heatmap of normal fecal samples showing clustering over time. R0011 = *L. rhamnosus* R0011; R0052 = *L. helveticus* R0052; SB = *S. boulardii* CNCM I-1079; R0175 = *B. longum* R0175; PROTO = ProtecFlor^TM^.


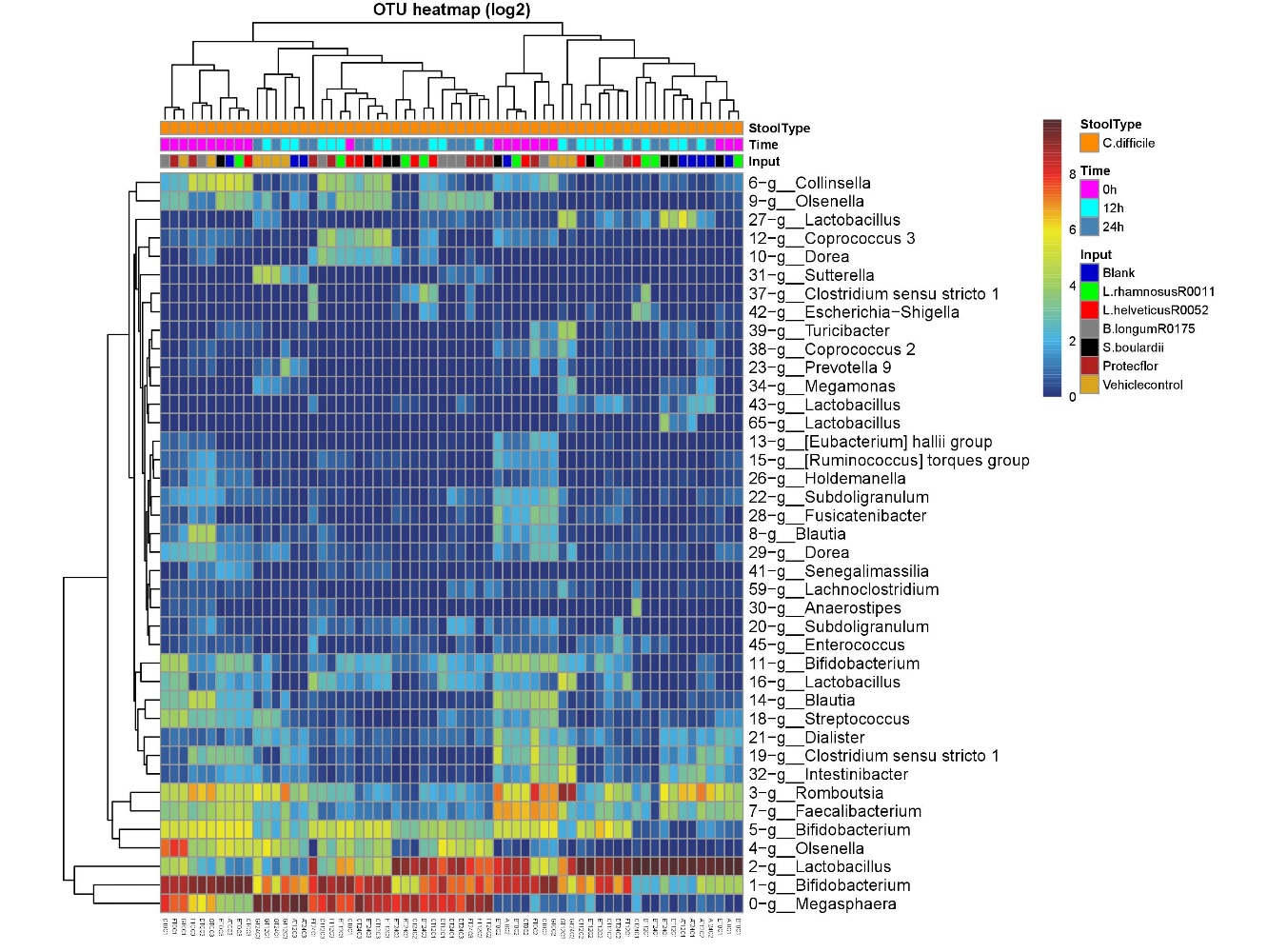


**Supplementary Figure S4.** Heatmap of operational taxonomic units (OTUs) of *Clostridium* (C.) *difficile*-infected fecal samples showing clustering over time. Values are shown in OTUs. R0011 = *L. rhamnosus* R0011; R0052 = *L. helveticus* R0052; SB = *S. boulardii* CNCM I-1079; R0175 = *B. longum* R0175; PROTO = ProtecFlor^TM^.


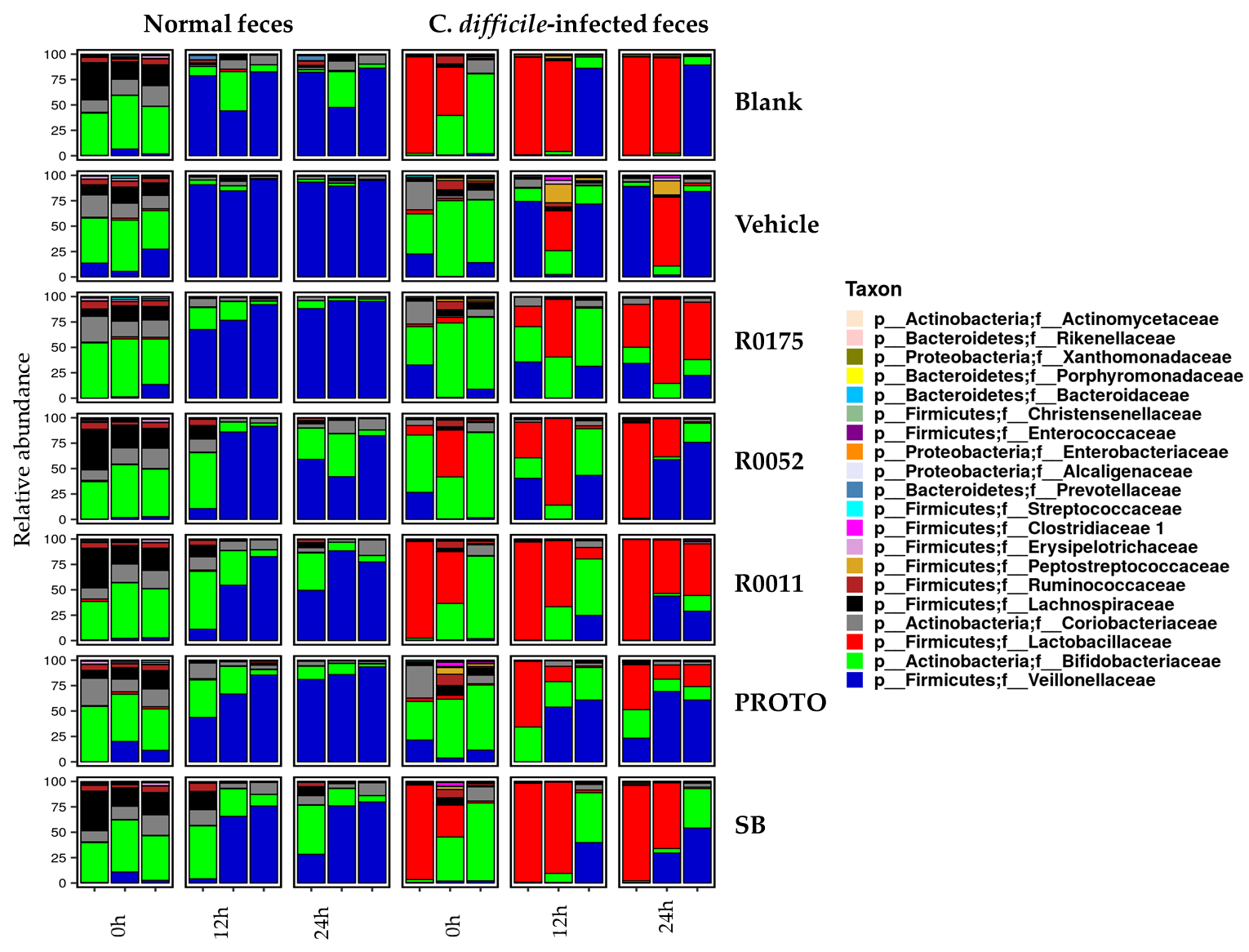


**Supplementary Figure S5.** Microbial diversity (family level) of normal feces, and, *Clostridium* (C.) *difficile*-infected feces showing relative abundance using amplicon sequence variants (ASVs). R0011 = *L. rhamnosus* R0011; R0052 = *L. helveticus* R0052; SB = *S. boulardii* CNCM I-1079; R0175 = *B. longum* R0175; PROTO = ProtecFlor^TM^.

| 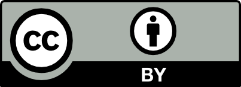 | © 2019 by the authors. Submitted for possible open access publication under the terms and conditions of the Creative Commons Attribution (CC BY) license (http://creativecommons.org/licenses/by/4.0/). |
| --- | --- |
