# Supplementary figures and images for: Probiotic Supplementation in a Clostridium difficile-Infected Gastrointestinal Model Is Associated with Restoring Metabolic Function of Microbiota
Source: Microorganisms. 2019 Dec 29;8(1):60. doi: 10.3390/microorganisms8010060 (PMC7023328; doi:10.3390/microorganisms8010060)

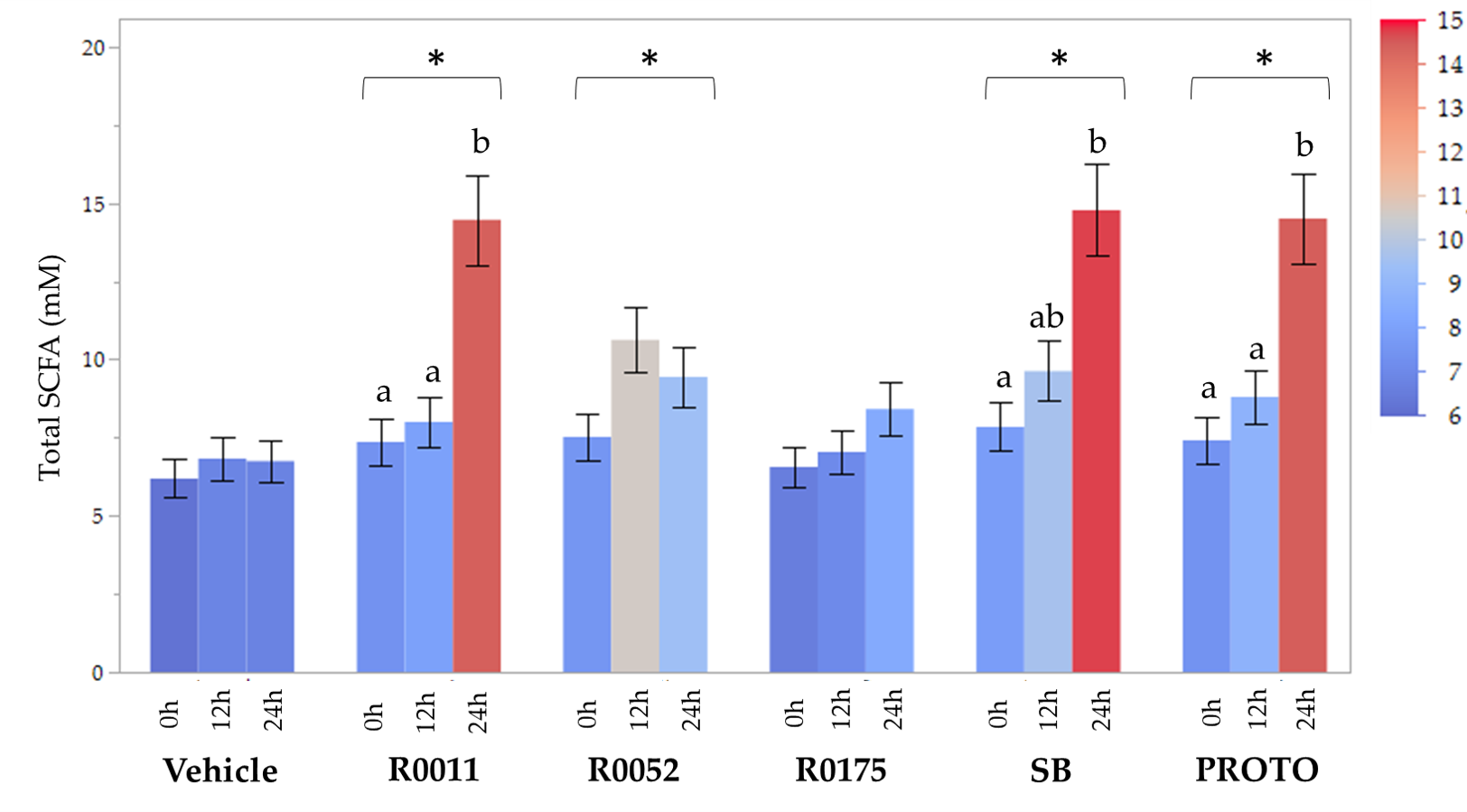

Supplement: Supplementary file 1 [file microorganisms-08-00060-s001.zip › Figure S1.png]

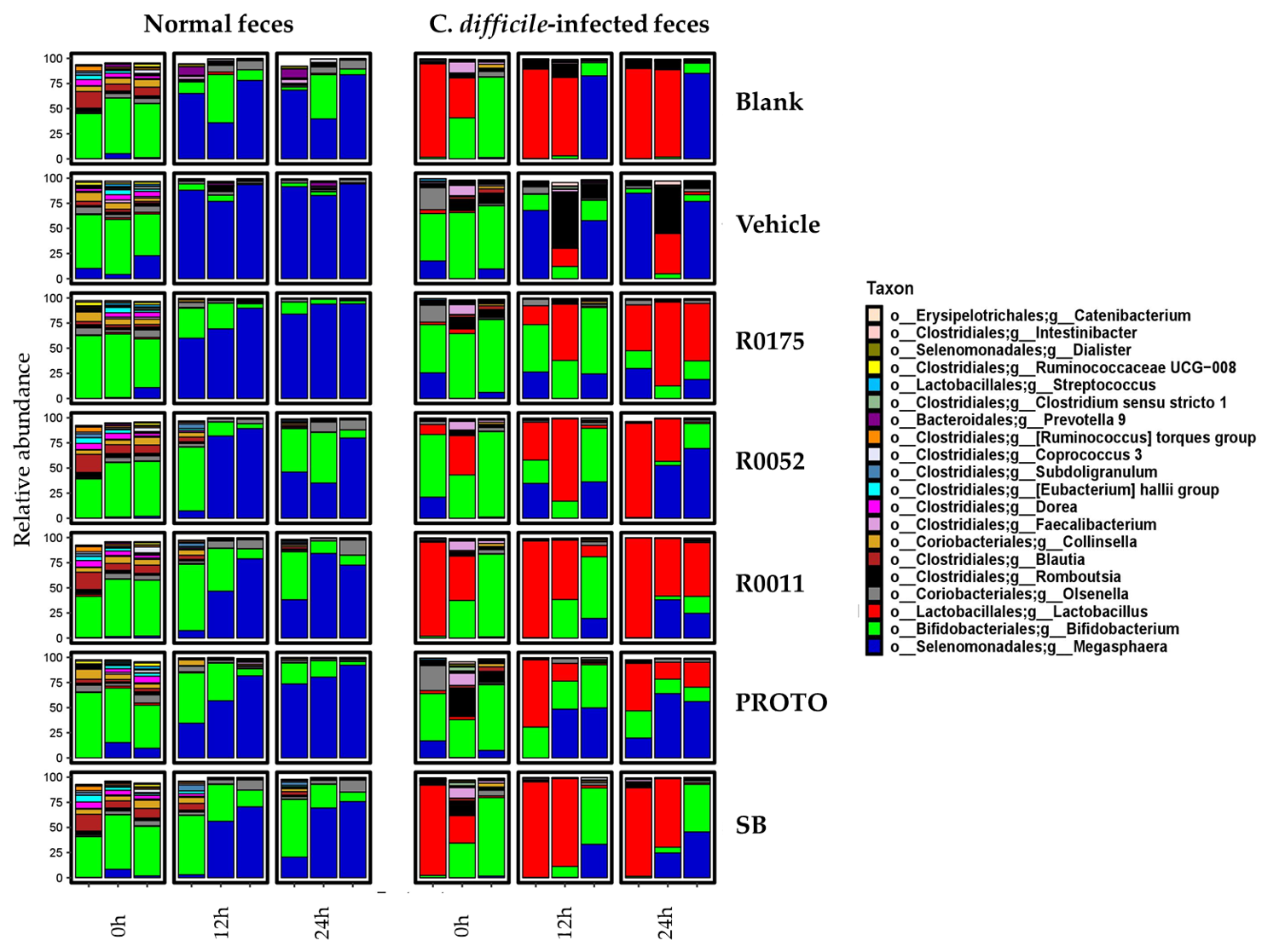

Supplement: Supplementary file 1 [file microorganisms-08-00060-s001.zip › Figure S2.png]

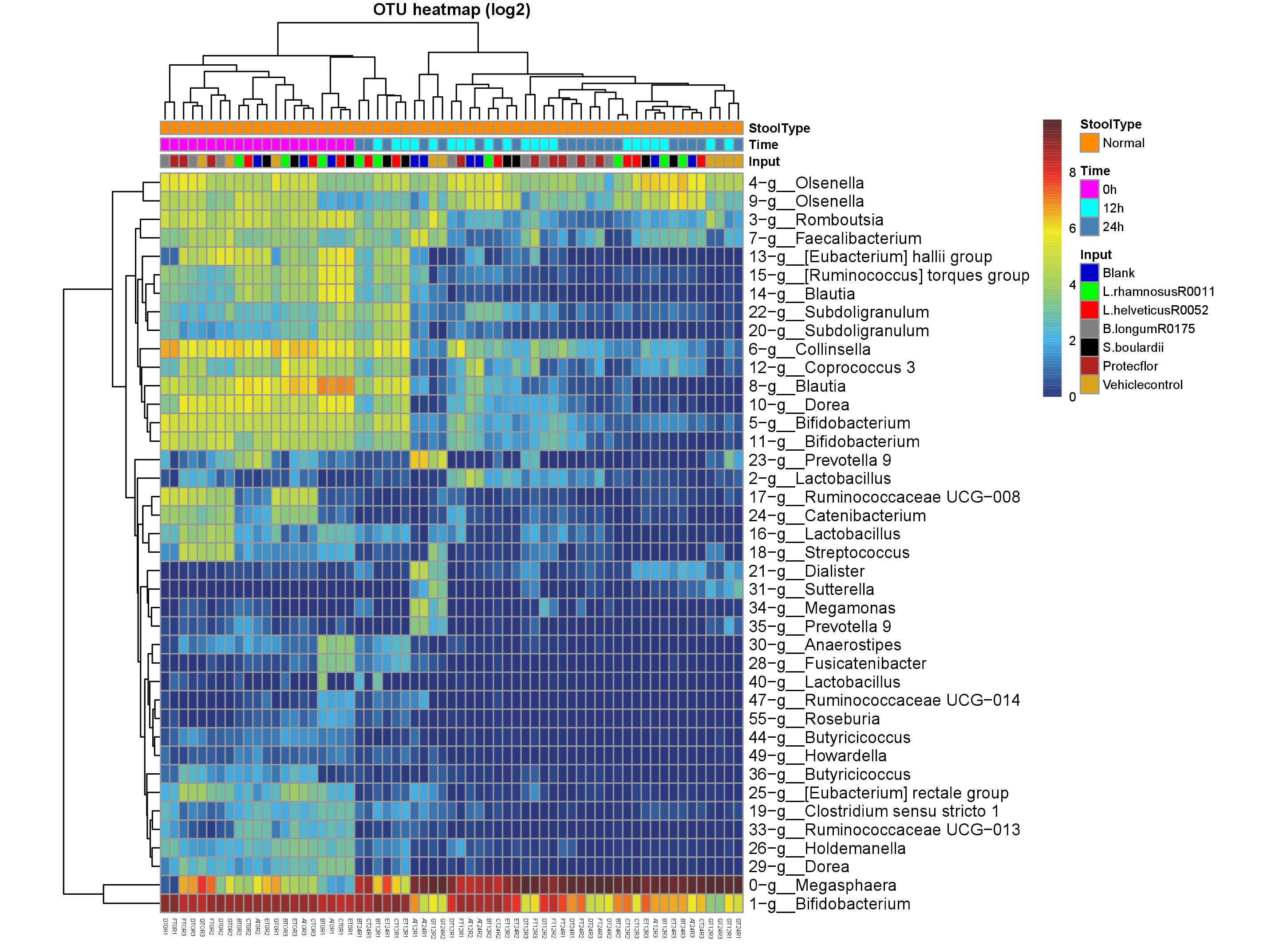

Supplement: Supplementary file 1 [file microorganisms-08-00060-s001.zip › Figure S3.jpg]

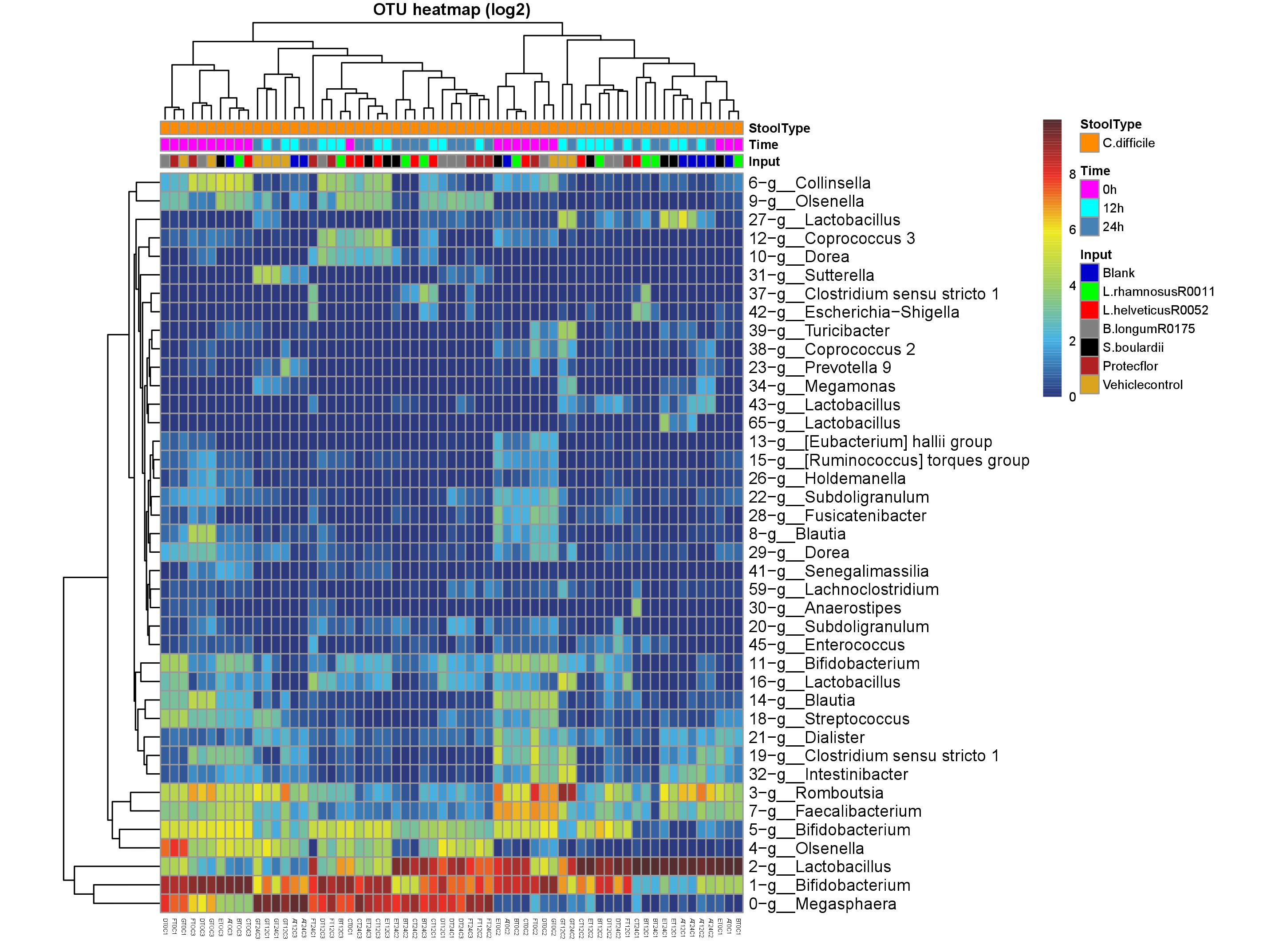

Supplement: Supplementary file 1 [file microorganisms-08-00060-s001.zip › Figure S4.jpg]

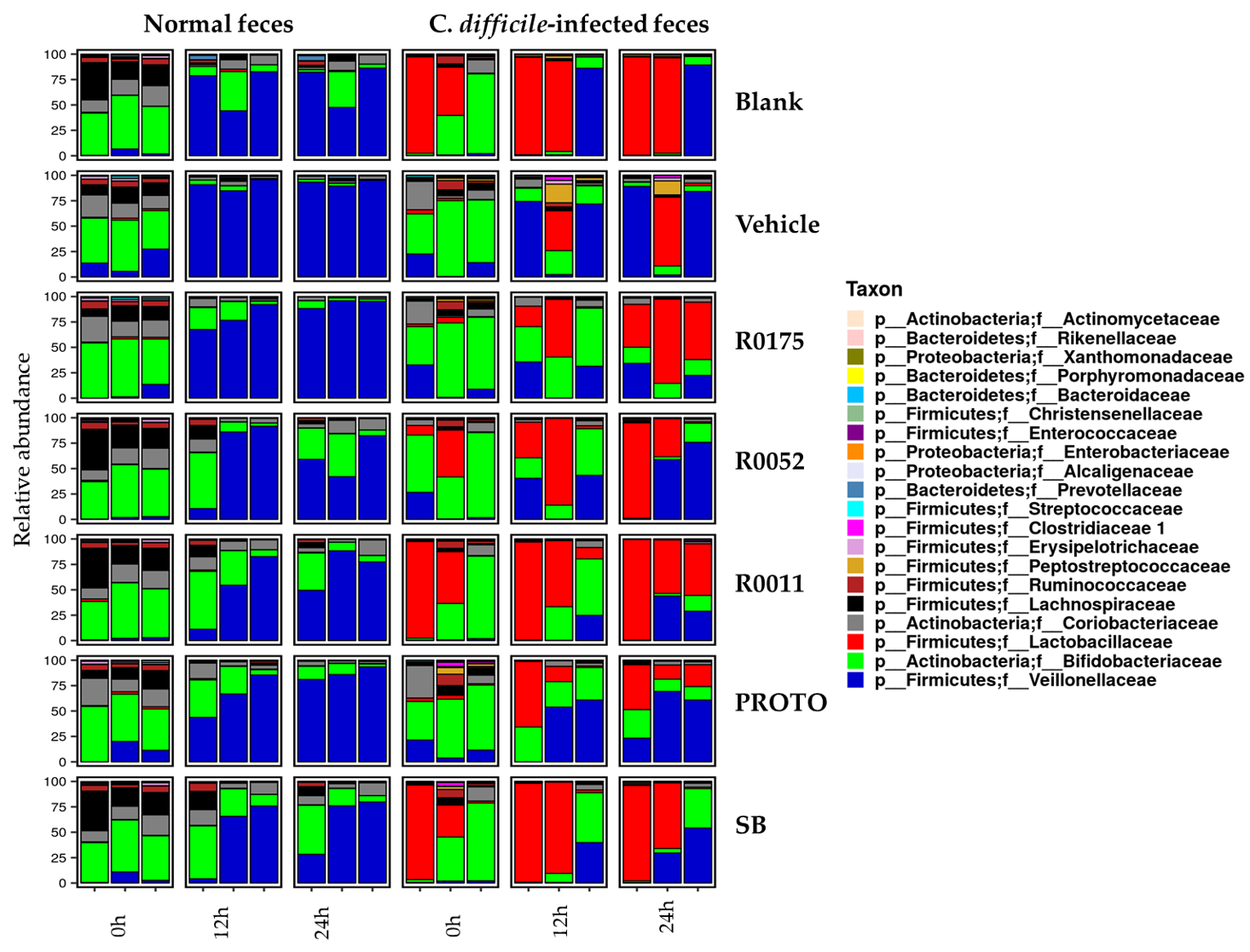

Supplement: Supplementary file 1 [file microorganisms-08-00060-s001.zip › Figure S5.png]
